# Late gadolinium enhancement of colorectal liver metastases post-chemotherapy is associated with tumour fibrosis and overall survival post-hepatectomy

**DOI:** 10.1007/s00330-018-5331-4

**Published:** 2018-02-23

**Authors:** Helen M. C. Cheung, Paul J. Karanicolas, Eugene Hsieh, Natalie Coburn, Tishan Maraj, Jin K. Kim, Howaida Elhakim, Masoom A. Haider, Calvin Law, Laurent Milot

**Affiliations:** 1 Department of Medical Imaging, Sunnybrook Health Sciences Centre, University of Toronto, 2075 Bayview Avenue, Rm AB 279, Toronto, ON Canada; 2 Department of Surgery, Sunnybrook Health Sciences Centre, University of Toronto, Toronto, ON Canada; 3 Department of Anatomic Pathology, Sunnybrook Health Sciences Centre, University of Toronto, Toronto, ON Canada

**Keywords:** Colorectal cancer, Neoplasm metastases, Gadolinium, Fibrosis, Survival

## Abstract

**Purpose:**

To determine whether late gadolinium MRI enhancement of colorectal liver metastases (CRCLM) post-chemotherapy is associated with tumour fibrosis and survival post-hepatectomy.

**Materials and methods:**

The institutional review board approved this retrospective cohort study and waived the requirement for informed consent. A cohort of 121 surgical patients who received preoperative MRI after chemotherapy between 2006-2012 was included in this study. Target tumour enhancement (TTE), defined as the mean contrast-to-noise ratio of up to two target lesions on late-phase gadobutrol-enhanced MRI, was determined by two independent raters. The average TTE was correlated with tumour fibrosis on post-hepatectomy specimens using Spearman correlation and with survival post-hepatectomy using Kaplan-Meier and Cox regression. Inter-rater reliability was determined using relative intra-class correlation coefficients.

**Results:**

In the surgical cohort (mean age: 63.0 years; male: 58%), TTE was associated with tumour fibrosis (*r* = 0.43, *p* < 0.001). Strong TTE was associated with improved survival compared to weak TTE (3-year survival: 88.4% vs. 58.8%, *p* = 0.003) with a hazard ratio of 0.32 (95% CI: 0.14-0.75, *p* = 0.008), after taking into account known prognostic variables. Inter-rater reliability was very good with a relative intraclass correlation of 0.84 (95% CI: 0.77-0.89).

**Conclusion:**

Late gadolinium MRI enhancement of CRCLM post-chemotherapy is associated with tumour fibrosis and survival.

**Key Points:**

*• MRI enhancement of colorectal liver metastases is associated with survival post-hepatectomy*

*• MRI enhancement of chemotherapy-treated colorectal liver metastases correlates with tumour fibrosis*

*• Measuring late MRI enhancement using target tumour enhancement is reliable*

## Introduction

Colorectal cancer is the second leading cause of cancer deaths in the developed world [1]. Approximately half of patients develop liver metastases and most deaths are related to metastatic disease [2]. The median survival of patients with colorectal liver metastases (CRCLM) without treatment is 7.5 months [3]. With advancements in surgical and chemotherapy techniques, the survival of patients with CRCLM has significantly improved. In a meta-analysis by Kanas et al. (2012), the 5- and 10-year survival of patients with resected CRCLM was 38% and 26%, respectively [2]. This is likely even higher with more recent data and improving surgical and chemotherapy techniques.

The ability to predict prognosis informs treatment recommendations, including surgery and/or chemotherapy. Several prognostic indicators stratify risk for patients with CRCLM including clinical, pathology, and molecular prognostic biomarkers (3-4). However, the use of magnetic resonance imaging (MRI) to stratify risk in patients with CRCLM is relatively unexplored. MRI is routinely used clinically for diagnosis, staging, and operative planning in patients being considered for liver resection, so information gained from MRI could be easily translated into clinical practice.

Several studies have demonstrated that tumour fibrosis in post-hepatectomy CRCLM specimens is associated with overall survival [4, 5]. This may be related to the pathological response to chemotherapy. Pathologically, tumour fibrosis in CRCLM closely resembles the appearance of tumour fibrosis in cholangiocarcinoma. In cholangiocarcinoma, late gadolinium enhancement on MRI with extracellular contrast agents is correlated with tumour fibrosis [6]. This association has also been reported with CRCLM, although this is less well studied [6]. Thus, we hypothesise that late gadolinium enhancement of colorectal cancer liver metastases may be correlated with tumour fibrosis post-chemotherapy and therefore with overall survival post-hepatectomy.

The purpose of our study was to determine whether late gadolinium enhancement of CRCLM on MRI with gadobutrol is associated with tumour fibrosis and overall survival post-hepatectomy.

## Materials and methods

This study was an institutional-REB approved, retrospective study.

### Participants

The retrospective cohort included all patients at a single tertiary cancer centre with CRCLM who had received a gadobutrol-enhanced MRI after treatment with chemotherapy (variable regimens as determined by standard of care, clinical treatment) and prior to hepatic resection for curative intent between January 1, 2006, and December 31, 2012. Preoperative MRI is performed as part of the routine imaging work-up for diagnosis and staging at this institution. All patients met institution guidelines for hepatic resection with curative intent (no extrahepatic disease at time of MRI) and were deemed fit for major surgery. The typical workflow in our institution is as follows: patients are referred to the hepatobiliary surgeons with outside ultrasound or CT imaging suggestive of CRCLM. Patients who are possible surgical candidates are then referred for MRI by the hepatobiliary surgeons for preoperative MRI prior to surgery.

Exclusion criteria included patients who did not have 10-min delayed-phase imaging, MRIs with image quality unacceptable for analysis, or patients that did not have measurable target lesions. Patients who died within 30 days of surgery were also excluded to eliminate deaths due to perioperative mortality. If multiple gadobutrol-enhanced MRIs were performed, the MRI closest to the surgical date was used for analysis.

Clinical and demographic information was obtained from electronic patient records as well as publicly available obituary databases, including age, sex, chemotherapy prior to MRI, and a validated clinical risk score, developed by Feroci and Fong [7]. The clinical risk score is calculated as a five-point scale, with one point for each of the following: number of tumours > 1, size of largest tumour ≥ 5 cm, metachronous metastases (time from diagnosis of primary to time of diagnosis of metastases ≤ 12 months), primary colorectal cancer with ≥ 5 lymph nodes positive, and preoperative carcinoembryonic antigen level ≥ 200 ng/ml [7]. A high preoperative clinical risk score is a validated predictor of poor long-term postoperative survival [7].

The clinical endpoint for this study was overall survival. Follow-up data were collected up to January 1, 2016.

### Magnetic resonance imaging (MRI) protocol and analysis

All patients received a gadobutrol-enhanced MRI for diagnostic and staging purposes as part of their routine clinical work-up using standard clinical liver imaging protocols at our institution. As part of the contrast-enhanced series, delayed 3D axial T1 imaging was routinely performed 10 min post-intravenous injection of gadobutrol (0.1 ml/kg body mass up to 10 ml at 1.0 mmol/ml). All studies were performed on 1.5-T (GE Twinspeed™, TR, 4.5; TE, 2.2; flip angle, 15; slice thickness, 5 mm; spacing, 2.5 mm; FOV, 380 mm; matrix, 320 × 192) or 3.0-T (Philips Achieva™, TR, 3.0; TE, 1.4; flip angle, 10; slice thickness, 3 mm; spacing, 1.5 mm, FOV, 380; matrix, 250 × 250) magnets with an eight-channel body phased array coil covering the entire liver.

Imaging analysis was performed on standard picture-archiving and communication system (PACS) software at our institution (Agfa Impax 6.3.1, AGFA HealthCare N.V., Belgium™). Up to two target lesions were identified as per Response Evaluation Criteria in Solid Tumours (RECIST) 1.1 criteria [8]. If multiple CRLMs met the criteria for target lesions, then the two largest measurable lesions were chosen. Patients were excluded from the study if there were no measurable lesions as defined by RECIST 1.1. Target lesions were confirmed as CRCLMs based on postoperative pathology reports.

For all target lesions, the contrast-to-noise ratio (CNR) on 10-min delayed phase was calculated using previously described methods [9]. At the axial level where the lesion was the largest, a round region of interest (ROI) most closely approximating the entire tumour was drawn to determine the lesion’s mean signal intensity (SI). The mean SI of five 1-2-cm ROIs drawn in the surrounding liver parenchyma (avoiding the tumour or major blood vessels) on the same slice as the tumour was determined. The standard deviation (SD) of the background noise was calculated from taking the mean SD of eight 1-2-cm ROIs drawn in the background in the four quadrants, taking care to exclude banding surrounding the patient due to motion artefact.

The CNR of each CRCLM was calculated as follows:

1 $$ CNR=\frac{SI\ (lesion)- SI\ (liver)}{SD\ (noise)}. $$

The target tumour enhancement (TTE) was calculated as the mean of the CNR of the target lesion(s). Two separate readers (HC and TM with 6 and 1 years of experience) independently determined the TTE. The mean TTE between the two readers was used for radiological-pathological and survival analysis.

### Pathology analysis

Gross tumour sizes were determined based on the largest diameter post-fixation (10% buffered formalin). Haematoxylin and eosin-stained slides were prepared from representative paraffin blocks. A single pathologist (HE, with 6 years of experience) qualitatively assessed the approximate percentage of fibrosis, necrosis, acellular mucin, and viable tumour cells on each representative slide. The CRCLMs identified on the pathology specimens were matched to the target CRCLMs identified on imaging by matching the location of the tumour and the size of the tumour. Patients were excluded from radiological-pathological analysis if the specimens were not available for analysis or if there were multiple CRCLMs of similar size in the same location that could not be matched on a per-lesion basis.

All imaging and pathology analyses were performed by readers blinded to all clinical information (other than the history of CRCLM).

### Statistical analysis

**Fig. 1:**
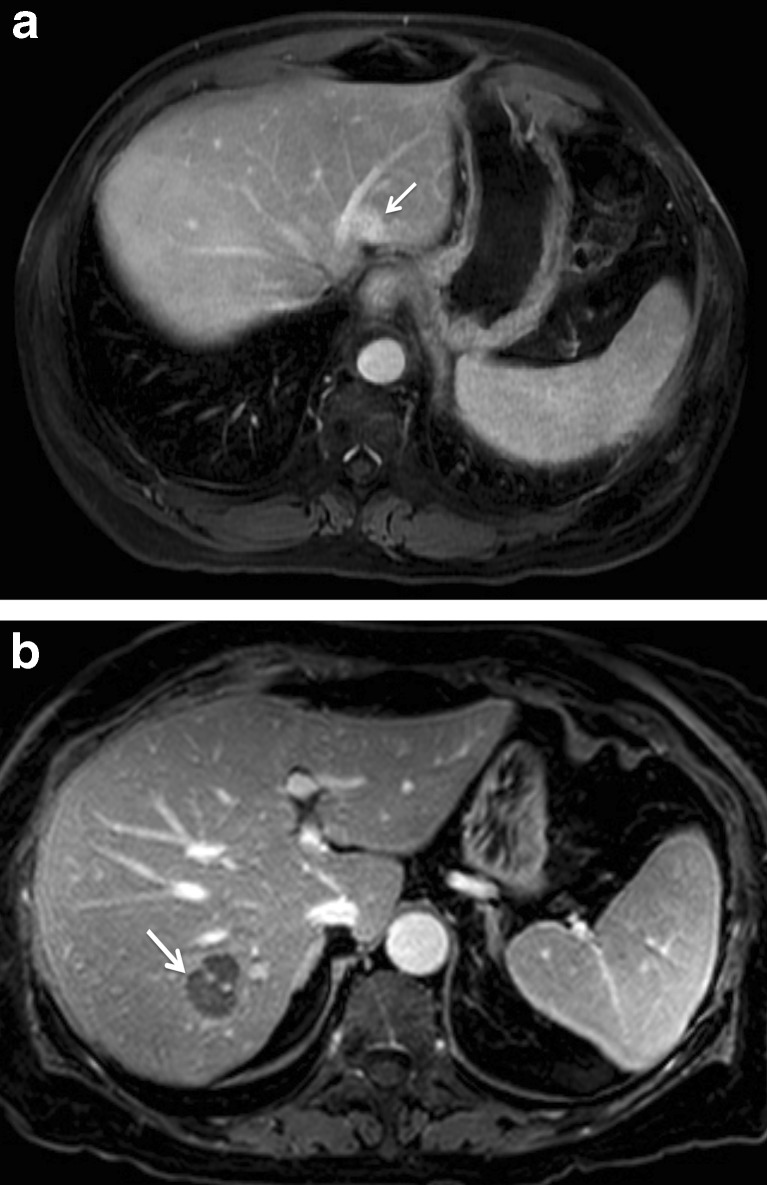
Colorectal liver metastases seen on 10-min delayed-phase, gadobutrol-enhanced MRI (**a**) in a 75-year-old male with strong target tumour enhancement and (**b**) in a 60-year-old male with weak target tumour enhancement

Patients were dichotomised into weak and strong TTE (Fig. 1). The cut-off point was determined using the surgical cohort using the Youden Index for 3-year survival [10].

A chi-Square test was used to determine if there were differences in demographic data between the strong and weak TTE groups.

Spearman correlations were used to determine whether there was a correlation between TTE and the mean percentage fibrosis, necrosis, acellular mucin, and viable tumour cells of the matched target lesions determined on pathological analysis. The median target percentage of fibrosis, necrosis, and viable tumour cells was determined for both high and low TTE.

The association between the patient’s TTE and survival was determined using Kaplan-Meier statistics. Multivariable Cox regression statistics were used to assess the association between TTE and survival after taking the clinical risk score into account.

Post-hoc sensitivity analyses were performed using Cox regression for time from MRI to surgery as well as for any demographic variables that demonstrated significant differences between strong and weak TTE (Table 1).

**Table 1:** Baseline demographics of patient population (*n* = 121, entire cohort)

	Weak target tumour enhancement (*n* = 74)	Strong target tumour enhancement (*n* = 47)	*p* value*
Age			
< 65 years	40 (54.1%)	24 (51.1%)	*p* = 0.75
≥ 65 years	34 (45.9%)	23 (48.9%)	
Sex			
Male	43 (58.1%)	27 (57.4%)	*p* = 0.94
Female	31 (41.9%)	20 (42.6%)	
Clinical risk score			
< 3	52 (76.5%)	34 (77.3%)	*p* = 0.92
≥ 3	16 (23.5%)	10 (22.7%)	
Number of tumours			
= 1 tumour	36 (48.6%)	17 (36.2%)	*p* = 0.18
> 1 tumour	38 (51.4%)	30 (63.8%)	
Tumour size			
< 5 cm	55 (74.3%)	43 (91.5%)	*p* = 0.019*
≥ 5 cm	19 (25.7%)	4 (8.5%)	
Time from diagnosis of primary to diagnosis of metastasis			
≤ 12 months	30 (40.5%)	13 (27.7%)	*p* = 0.149
> 12 months	44 (59.5%)	34 (72.3%)	
Number of positive lymph nodes			
< 5 nodes positive	53 (73.6%)	36 (76.6%)	*p* = 0.71
≥ 5 nodes positive	19 (26.4%)	11 (23.4%)	
Data not available	2	0	
Preoperative CEA level			
< 200 ng/ml	60 (95.2%)	43 (95.6%)	*p* = 0.94
≥ 200 ng/ml	3 (4.8%)	2 (4.4%)	
Data not available	11	2	
Magnet			
1.5 T	45 (60.8%)	30 (63.8%)	*p* = 0.74
3.0 T	29 (39.2%)	17 (36.2%)	

Additional post-hoc analyses were also performed to determine the proportion of patients with heterogeneous target lesions (1 lesion with CNR < 11 and 1 lesion with CNR > 11). Sensitivity analysis was performed excluding patients with heterogeneous target lesions to determine whether heterogeneity affected our results.

The TTE determined by each rater was compared using relative intra-class correlation coefficients to determine inter-rater reliability (two-way mixed model).

## Results

**Fig. 2:**
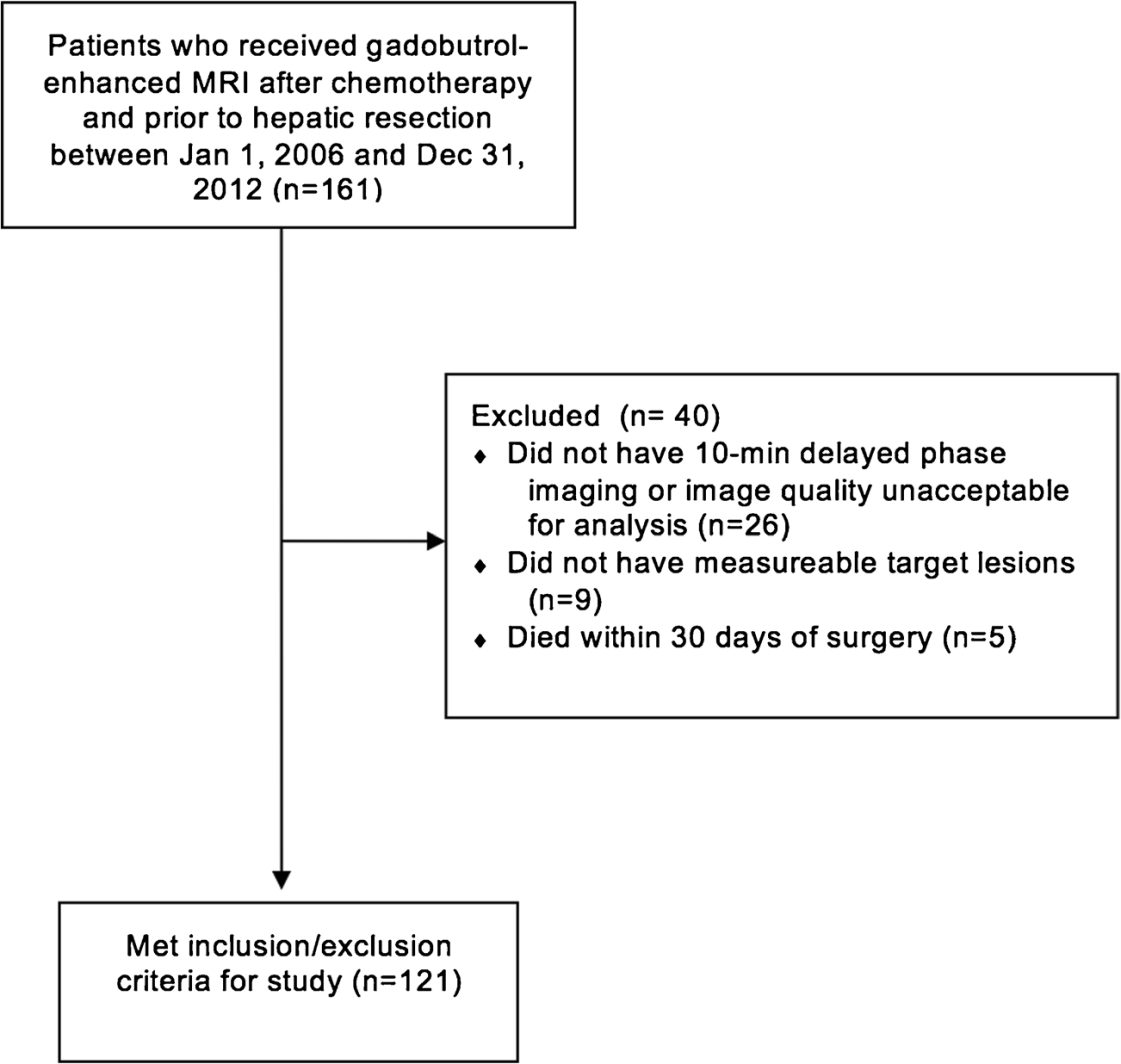
Flow charts of inclusion and exclusion criteria

Among the 121 patients who met inclusion/exclusion criteria for the study (Fig. 2), the mean age was 63.0 years (SD: 11.2 years) with 70 (57.9%) males and 51 (42.1%) females (Table 1). The median time from MRI to surgery was 2.7 months (range: 0.1-10.5 months). There were 40 deaths during the follow-up period.

Based on the Youden Index, the optimal cut-off for weak and strong TTE was CNR = 11. Seventy-four patients (61.1%) had weak and 47 patients (38.8%) strong TTE.

Patients with strong TTE were more likely to have smaller tumours (*p* = 0.019). No other demographic data were significantly different between the MRI groups (Table 1).

**Fig. 3:**
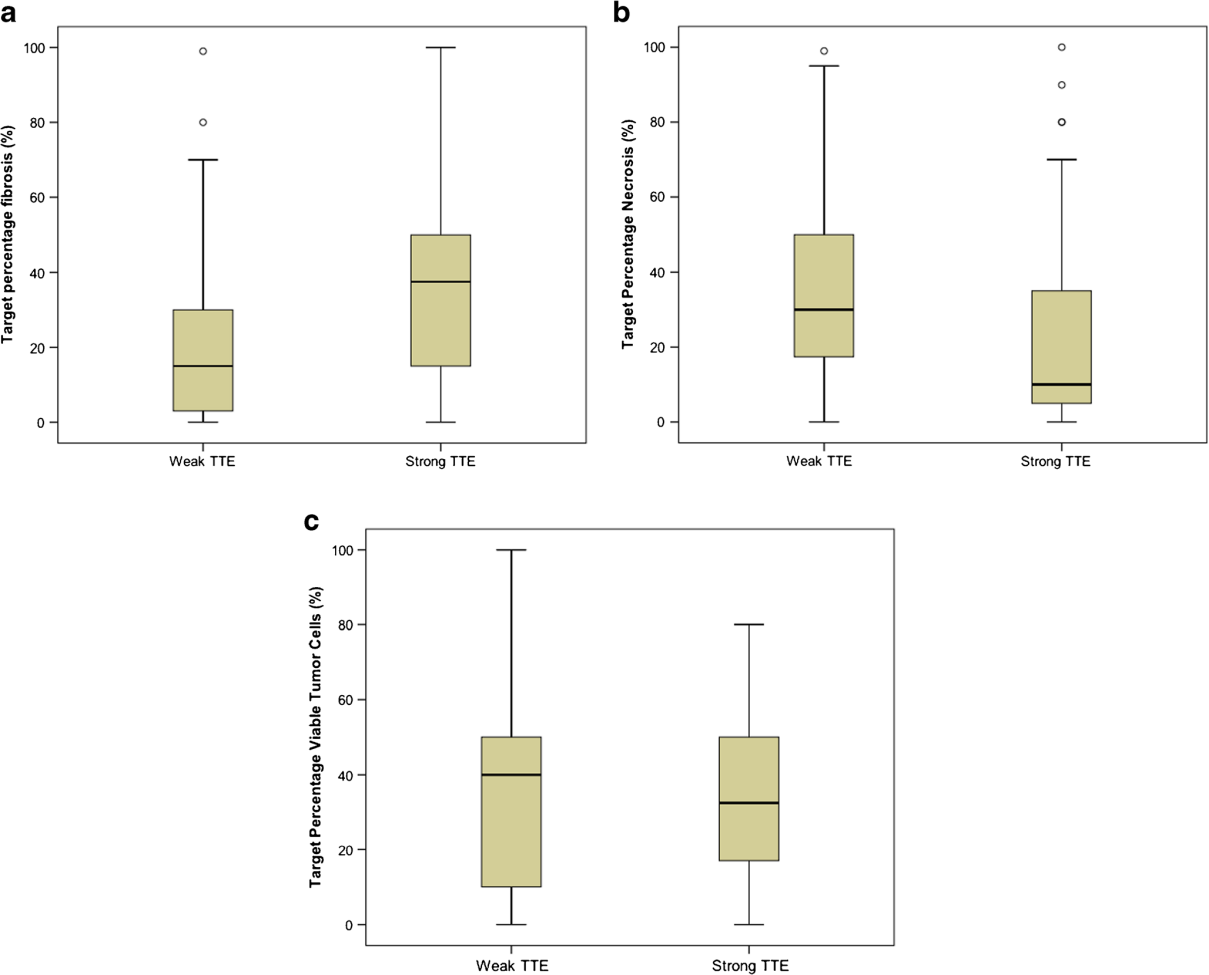
Boxplots demonstrating median target percentage (**a**) fibrosis, (**b**) necrosis, and (**c**) viable tumour cells among patients with strong and weak TTE (*n* = 91, for histological analysis)

It was possible to accurately match lesions between MRI and pathology for 91 patients with 126 target CRCLMs. The Spearman correlations between TTE and the mean target percentage of fibrosis, necrosis, acellular mucin, and viable tumour cells were 0.43 (*p* < 0.001), -0.22 (*p* = 0.036), 0.02 (*p* = 0.84), and -0.05 (*p* = 0.63), respectively. The median target percentage of fibrosis for high TTE and low TTE were 15.0% [interquartile range (IQR): 3.0% to 30.0%] and 37.5% (IQR: 15.0% to 51.3%), respectively (Fig. 3a). The median target percentage necrosis for high TTE and low TTE was 30.0% (IQR: 15.0% to 50.0%) and 10.0% (IQR: 3.8% to 35.0%), respectively (Fig. 3b). The median target percentage viable tumour cells for high and low TTE was 40.0% (IQR: 10.0% to 50.0%) and 32.5% (IQR: 16.5% to 50.0%) respectively (Fig. 3c). Most patients did not have tumours that contained acellular mucin (only 12 patients); therefore, the median target percentage acellular mucin was 0% for both high and low TTE.

**Fig. 4:**
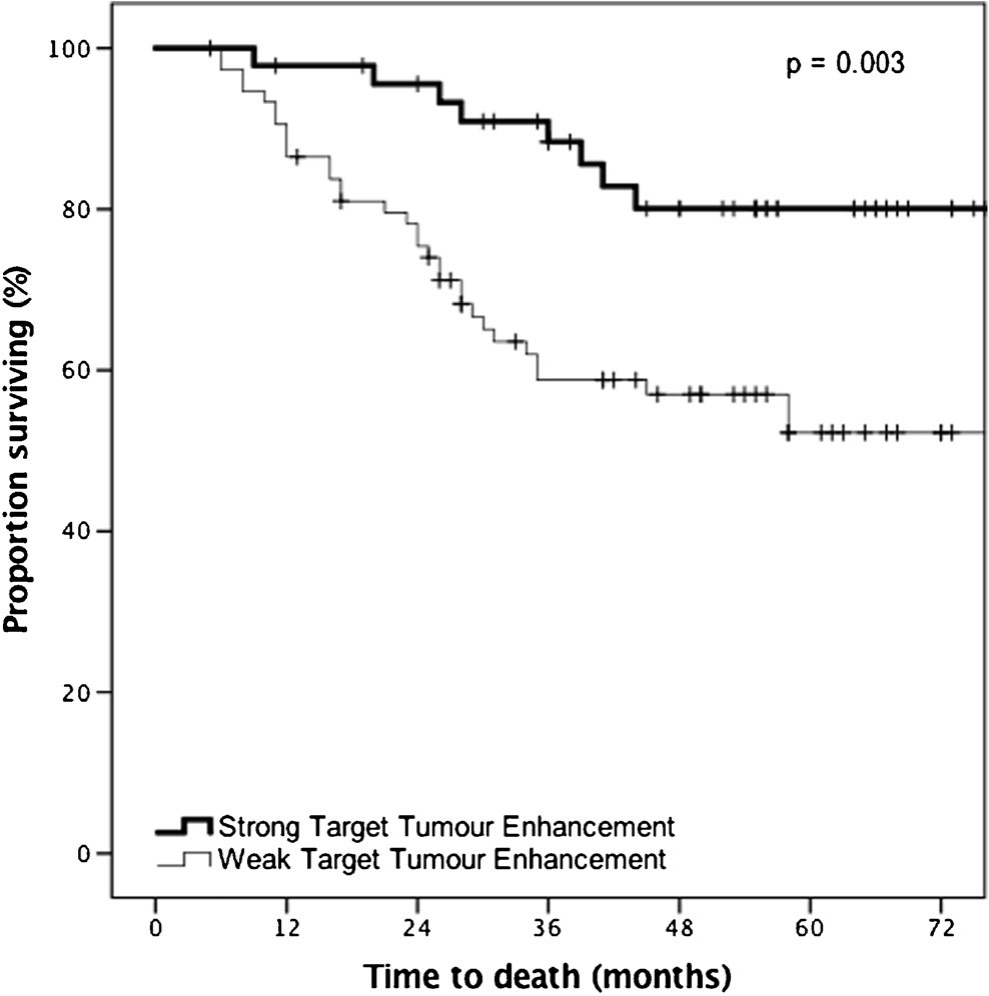
Kaplan-Meier survival curves showing the association between target tumour enhancement of colorectal liver metastases post-chemotherapy and overall survival in patients who received a gadobutrol-enhanced MRI prior to liver resection (*n* = 121, for univariate analysis)

Strong TTE was associated with survival on univariate analysis (*p* = 0.003). At 3 years, 88.4% of patients with strong TTE on the preoperative MRI were alive vs. 58.8% in patients with weak TTE (Fig. 4).

One hundred twelve patients (with 34 events) had complete data available for the multivariable analysis. TTE had an adjusted hazard ratio of 0.32 (95% CI: 0.14-0.75, *p* = 0.008). The adjusted hazard ratio of the clinical risk score was 2.41 (95% CI: 1.19-4.90) (Table 2).

**Table 2:** Cox regression model of surgical cohort for the association of target tumour enhancement (TTE) and overall survival (*n* = 112, for multivariate analysis)

	Adjusted odds ratio (95% confidence interval)	*p* value
Target tumour enhancement		
Weak	Reference	*p* = 0.008**
Strong	0.32 (0.14-0.75)	
Clinical risk score		
< 3	Reference	*p* = 0.015*
≥ 3	2.41 (1.19-4.90)	

Post-hoc sensitivity analyses were performed for time from MRI to surgery and tumour size ≥ 5 cm. None of these variables were found to be significant contributing variables on our sensitivity analysis. When time from MRI to surgery was included in the Cox regression model, TTE had an adjusted hazard ratio of 0.33 (95% CI: 0.14-0.75, *p* = 0.009). When tumour size was included in the Cox regression model, TTE had an adjusted hazard ratio of 0.33 (95% CI: 0.14 to 0.77, *p* = 0.010).

For reader 1, 62 patients (51.2%) had only 1 target lesion, 45 patients (37.2%) had 2 target lesions with homogeneous CNR (CNR < 11 for both lesions or CNR > 11 for both lesions), and 14 patients (11.6%) had 2 target lesions with heterogeneous CNR (1 lesion with CNR < 11 and 1 lesion with CNR > 11). When the 14 patients with heterogeneous target lesions were excluded, there was no significant difference in our results with TTE having an adjusted hazard ratio of 0.36 (95% CI: 0.15 to 0.90, *p* = 0.029).

For reader 2, 58 patients (47.9%) had only 1 target lesion, 45 patients (37.2%) had 2 target lesions with homogeneous CNR (CNR < 11 for both lesions or CNR > 11 for both lesions), and 18 patients (14.9%) had 2 target lesions with heterogeneous CNR (1 lesion with CNR < 11 and 1 lesion with CNR > 11). When the 18 patients with heterogeneous target lesions were excluded, there was no significant difference in our results with TTE having an adjusted hazard ratio of 0.29 (95% CI: 0.11 to 0.76, *p* = 0.012).

Inter-rater reliability was very good with a relative intraclass correlation of 0.84 (95% CI: 0.77-0.89).

## Discussion

In this study, we demonstrated that late gadolinium enhancement of CRCLM post-chemotherapy on gadobutrol-enhanced MRI post-hepatectomy is associated with tumour fibrosis and with overall survival, after taking into account known clinical prognostic factors. The absolute difference in 3-year survival was 29.6% less in patients who had weak TTE than in those who had strong TTE on preoperative MRI, with an adjusted hazard ratio of 0.32.

TTE on preoperative MRI was positively correlated with tumour fibrosis and negatively correlated with tumour necrosis on post-hepatectomy specimens, which may be the physiological explanation for this MRI phenomenon. No prior studies have specifically correlated the late gadolinium enhancement in CRCLM with tumour fibrosis, although studies have looked at the correlation between noncontrast MRI signal characteristics of CRCLM and tumour fibrosis [11]. It is well established in the pathology literature that tumour fibrosis in CRCLM is one of the major pathological responses to chemotherapy and the predominant pathological response associated with treatment response and long-term outcomes [4, 5, 12]. Specifically, tumour fibrosis and not tumour necrosis post-chemotherapy is associated with good long-term prognosis [4]. Tumour necrosis is known to be poorly enhancing on contrast-enhanced MRI, which could be a confounding factor for measurement of TTE [13]. In addition, tumour necrosis would be inversely correlated with tumour fibrosis since these variables may demonstrate collinearity.

Patients with strong TTE were more likely to have smaller tumours (*p* = 0.019). If strong TTE represents “good” biology, then these tumours may be less aggressive and therefore tend to be smaller. However, tumour size was not a confounding variable in our post-hoc sensitivity analyses, which suggests that TTE may reflect “good” biology independent of tumour size.

RECIST is the most commonly used technique for evaluation of chemotherapy response [8]. However, it is a size-based technique that has been shown to correlate poorly with pathological response or long-term survival [14]. Several imaging criteria have been developed to address these limitations, including CT-based morphological criteria, which showed good association with pathological response and survival in the setting of CRCLM treated with bevacizumab-containing chemotherapy [15]. Some authors have assessed the role of imaging techniques in assessing tumour biology, such as DCE-MRI and PET, although these techniques are expensive and time-consuming and are less routinely performed in the clinical setting [15–18].

Our study had several limitations, mostly related to its retrospective nature. There was variability in the timing of MRI in relationship to the administration of chemotherapy and the type of chemotherapy administered. Radiological-pathological correlation was limited by sampling error, which could decrease Spearman correlation particularly in tumours with significant heterogeneity, and not all lesions could be matched on a per-lesion basis, which could lead to selection bias. Additionally, tumour fibrosis can be seen in CRLM even in patients without chemotherapy [19]. These confounding factors may contribute to the relatively weak correlation between tumour fibrosis and TTE observed in our study.

Although 10-min delayed-phase imaging is part of our institution’s routine clinical liver MRI protocol, the addition of a 10-min delayed phase scan may impede workflow and be a limitation at institutions that only perform imaging to 3- or 5-min post-contrast. We performed TTE analysis at 10-min delayed phase based on the cardiac MRI literature, which has shown that fibrosis is best seen between 10 and 30 min [20, 21]. However, it is unclear whether this is also the case for tumour fibrosis within CRCLM and further studies should be performed to determine whether a 3-5-min delay is also sufficient.

Because MRIs were not obtained for the purpose of measuring CNR, several technical confounders including magnetic field strength, use of phased-array surface coils, and presence of diffuse liver disease may have affected our results [22, 23].

Our study, while important, demonstrates the need for additional prospective studies to confirm the results, for external validation, and to determine its potential clinical impact. Prospective studies are also required to determine the role of specific chemotherapy regimens analysing pre- and post-treatment MRI scans, to confirm correlation between MRI signal and fibrosis using registered, high-resolution, radiological-pathological techniques, to optimise selection of target lesions, and to optimise measurement of TTE through T1 signal mapping. In some patients with multiple CRCLMs, the enhancement pattern of different lesions can be heterogeneous. The presence of heterogeneous target lesions did not affect our results on our post-hoc sensitivity analysis, likely because of the relatively small proportion of patients with heterogeneous target lesions (approximately 12-15% of our patient cohort). However, further studies are required to determine the optimal method of measuring TTE in these patients. Although reliability between raters in our study was very good, development of standardised semi-automated techniques may further improve reliability.

As many patients are now staged using MRI with hepatobiliary-specific contrast agents, investigating the relationship between late-phase enhancement of CRCLM and tumour fibrosis and survival may also be valuable [24].

In conclusion, this article presents the first study to provide evidence that late gadolinium MRI enhancement of tumours post-chemotherapy is associated with tumour fibrosis and overall survival post-hepatectomy in patients with CRCLM. Target tumour enhancement on MRI may be a useful tool for risk-stratification. Further studies are required for external validation and to assess its potential clinical impact.

## Abbreviations

CNR: Contrast-to-noise ratio
CRCLM: Colorectal liver metastases
DCE-MRI: Dynamic contrast-enhanced MRI
MRI: Magnetic resonance imaging
PET: Positron emission tomography
RECIST: Response evaluation criteria in solid tumours
ROI: Region of interest
SD: Standard deviation
SI: Signal intensity
TTE: Target tumour enhancement
